# Whose story is it? Mental health consumer and carer views on carer participation in research

**DOI:** 10.1111/hex.12954

**Published:** 2019-08-28

**Authors:** Alyssa R. Morse, Owen Forbes, Bethany A. Jones, Amelia Gulliver, Michelle Banfield

**Affiliations:** ^1^ ACACIA: The ACT Consumer and Carer Mental Health Research Unit, Centre for Mental Health Research, Research School of Population Health The Australian National University Canberra Australian Capital Territory Australia

**Keywords:** caregivers, carers, consumer involvement, consumer participation, ethics, mental health, research ethics

## Abstract

**Background:**

Mental health carers contribute a unique set of perspectives and lived experiences to research; however, national research ethics guidelines do not specifically address the issues that affect informal carers as participants.

**Objective:**

This study sought to explore Australian mental health consumer and carer views on the ethical conduct of research involving mental health carers.

**Design:**

A public forum (n = 14; consumer = 5, carer = 9) and a subsequent series of interviews (n = 10; consumer = 5, carer = 4, both = 1) were conducted to investigate consumer and carer views on mental health research ethics. Data collection and analysis drew strongly on methodological features of grounded theory.

**Results:**

Conducting research involving carers and consumer‐carer relationships raises potential concerns related to story ownership. Lived experience stories have shared and separate elements; thus, it is important to consider potential risks to the privacy of non‐participants and of social harm to participants' relationships when conducting research in this space. These risks could be minimized and managed through communication between researchers and participants, and within relationships.

**Conclusions:**

When conducting research involving carers and consumer‐carer relationships, researchers may need to facilitate the negotiation of information‐sharing boundaries within relationships and the safe and confidential telling of shared stories.

## 1 INTRODUCTION

Australian government policies emphasize a key role for both consumers and carers in the development of mental health services and policy.^1^, ^2^ However, mental health policies often do not clearly distinguish between the needs of consumers and carers, suggesting their needs are similar or can be met through similar initiatives.^1^, ^2^, ^3^ Carer participation in research has increased in recent years, spanning areas including lived experiences of caring in different populations,^4^, ^5^ interactions with health and mental health services,^6^, ^7^, ^8^ support services for carers 9 and the active involvement of carers in research.^10^ Carers contribute a unique set of perspectives, lived experiences and agendas,^11^, ^12^, ^13^, ^14^, ^15^ and evidence from the literature suggests that consumers and carers encounter different challenges when they are involved in research, both as active researchers 11, 12 and as participants.^13^, ^14^ Therefore, it is important to develop clear guidelines to support the ethical and safe conduct of research that considers issues important to this population.

Australian, United States and Canadian national research ethics guidelines do not provide specific guidance for research with carer participants (i.e. unpaid friends or family members who support a person with mental illness).^16^, ^17^, ^18^ Thus, general ethics principles and guidelines must be applied when working with this population. A particular area of concern is story ownership: carers have their own unique experiences and a right to participate in research exploring those experiences,^19^ but in some cases there may be a perception of interconnectedness with the consumer's story and this raises the possibility of tensions over privacy. This situation places participants at risk of harm as defined by the Australian National Statement on Ethical Conduct in Human Research.^18^ Where tensions over privacy arise, participants may be at risk of social harm 18; participating in research could damage a carer's relationship with the person they care for. When stories are interconnected, non‐participants (including consumers) may be at risk of having sensitive personal information shared and subsequently identified by a third party. Evidence is required to determine the likelihood and severity of these risks, and how to minimize and manage them in a manner that is effective and acceptable for mental health carers and consumers.^18^, ^20^

A small number of best practice guides and examples are available to assist with this process. The UK National Institute for Health Research Mental Health Research Network (MHRN) commissioned a good practice guide for involving carers in mental health research,^19^ which includes a brief description of carers' rights, stating that carers have their own rights, experiences and stories to tell. The guide also highlights the role that carers can play in using their lived experience to develop ethics guidelines and ethical research projects.^19^ An equivalent Australian good practice guide is not currently available. A similar approach to mental health carer research design was described by Allam and colleagues 11 in their report of an outreach service evaluation conducted collaboratively by consumer and carer researchers. Interviews with carers were designed to focus on the support the service had provided to carers and avoid focusing on the experiences of the person for whom they cared.^11^ These examples provide some guidance for managing the potential social and non‐participant harms of carer‐focused research, but further evidence is needed to assist with gauging risk levels and to determine which management approaches are acceptable to carers and consumers. The perspectives of people with lived experience of mental illness, as a consumer or carer, can inform these ethical decisions.^20^, ^21^, ^22^

The current research aimed to explore Australian consumer and carer views on the ethical conduct of research involving mental health carers. The research also aimed to contribute to the development of practical guidance, consistent with and supplementary to the National Statement,^18^ to support researchers working with carer participants. The focus of the investigation was to explore issues related to story ownership and the consumer‐carer relationship.

## 2 METHODS

### 2.1 Ethics approval

The ethical aspects of the project were approved by The Australian National University Human Research Ethics Committee (protocol number 2015/247). All participants were required to read an information sheet and give written consent before participating. Interview participants were offered a $20AUD voucher for their time.

### 2.2 Design

This consumer‐led research project was conducted at ACACIA: The ACT Consumer and Carer Mental Health Research Unit. All researchers at ACACIA have personal lived experience with mental health issues, as a consumer and/or a carer, and use both their personal lived experience and their academic training to inform their research practice. The approach to data collection and analysis drew strongly on the methodological features of grounded theory.^23^ The first stage of the research was a public forum held with consumers and carers to gain their perspectives on research ethics and participation. The forum was relatively unstructured and analysed to create an initial framework of themes. An interview procedure was subsequently developed from the framework to further explore these themes with consumers and carers. Theoretical sampling was employed during interview recruitment to explore identified concepts and seek out potentially explanatory or contradictory information. The interview data were used to test and modify the thematic framework. The ACACIA Consumer and Carer Advisory Group collaborated on the structure, organization and focus of the study, including protocols and questions.

### 2.3 Forum procedure

ACACIA held a half‐day forum in June 2015. The forum was advertised as part of ACACIA's research and recruitment flyers clearly indicated that all attendees would be research participants. Recruitment materials used the headline ‘Whose story is it?’ and included the prompts: ‘Can consumers and carers participate in research if the other declines? How can carers of people who disagree with their diagnosis participate in research? Can consumers and carers participate without the knowledge of the other?’ These questions were selected to stimulate discussion about situations that could lead to ethical dilemmas in a research context.

All forum attendees participated in the research. Fourteen people with lived experience of mental illness, either as a consumer (n = 5) or as a carer (n = 9), were recruited through local mental health consumer and carer organizations and the register of people interested in the work of ACACIA. Five participants (including author MB) were also lived experience researchers (i.e. researchers with personal experience as a mental health consumer and/or carer) who could bridge the consumer/carer and research perspectives and facilitate discussions.^24^ Lived experience researchers use their personal experience of mental illness and their academic training to inform their research practice. It was valuable to collect the perspectives of this group as their views may differ from those of consumers and carers without formal training.^24^ The forum was facilitated by a professional mental health advocate and peer trainer, who identified as a consumer, and had significant previous experience facilitating events with a mental health focus. The facilitator briefed participants regarding respectful communication, safety and self‐care. Discussions were initially separated into consumer‐only and carer‐only groups, after which consumers and carers were brought together for a combined discussion of the issues.

Forum recordings were transcribed verbatim, and initial analyses were conducted on de‐identified transcripts by researchers who did not participate in the forum event to reduce the opportunity for bias. The coding framework was developed by one author (BAJ). This framework was subsequently refined by a second author (OF) and reviewed by the other authors, including the author present at the forum, to produce the final themes and associated quotes.

### 2.4 Interview procedure

Forum findings provided the basis for the topic guide for the interviews, with the aim of building on the initial results. Consistent with the principles of grounded theory,^23^ the interviews sought to develop a deeper understanding of concepts that had emerged during the forum discussions but were not explored in depth and investigate whether strongly contrasting consumer and carer views had been amplified by in‐group identification at the forum. Interview questions were deliberately neutrally worded and participants were asked to consider hypothetical scenarios to encourage them to think reflectively and consider multiple perspectives.

Ten participants (nine female and one male) were recruited for the interviews. Five identified as consumers, four as carers, and one as both a consumer and a carer. Five participants (three carers and two consumers) were initially recruited using advertising as described for the forum. Theoretical sampling was then employed to develop a deeper understanding of several concepts. Carer participants at the forum and in the early interviews had provided high levels of support for a family member; in contrast, most consumer forum participants received occasional social support from family and friends. This mismatch in experience may have driven some of the contrasting views on story ownership. To address this, carers who had provided occasional support for a friend or family member were interviewed to explore for alternate views. As many previous participants had been engaged in mental health advocacy or education, it was important to explore whether this affected their views on storytelling and research practice. Young adult consumers and carers with little or no advocacy or representation experience were interviewed to explore this area. Data from all conducted interviews were included in the analysis.

Approximately one‐hour‐long interviews were conducted in‐person by one of two authors (ARM or OF) and audio‐recorded with participants' consent. At the beginning of each interview, participants were given a description of the aim of the research project, the purpose of the interview and a definition of the term ‘carer’, to ensure a shared understanding. After each interview, interviewers recorded written reflective notes about the nature and key content of the discussion. Audio‐recordings were transcribed verbatim with identifying information removed. Analyses were performed on the transcripts and interviewer reflective notes.

Interview analysis was conducted by one author (ARM), in consultation with the research team. Data were managed using QSR International's NVivo 11 Software. An initial coding framework was developed using the themes and subthemes from the forum analysis and key concepts identified from interviewers' reflective notes. As coding progressed, the applicability of the framework was tested and themes were modified to accommodate new information. Memos were used to facilitate and record the process of developing the final thematic framework. Throughout this process, the coding framework and thematic development were regularly discussed with other members of the research team to test assumptions and clarify themes.

## 3 RESULTS

Two major themes related to the ethical conduct of research involving mental health carers were identified in the final framework. These themes were *ownership of story* and *communication and education*, and their content was primarily concerned with the consumer‐carer relationship and the responsibilities of researchers regarding these relationships. The final framework included five additional themes related to general procedural ethics considerations for mental health research: these themes are discussed in a separate paper.^25^

### 3.1 Ownership of story

*Ownership of story* received the most discussion and was expressed differently across the three forum sessions. The consumer group emphasized that consumers can have a greater degree of vulnerability in the consumer‐carer relationship, so they should also have greater ownership and control over what happens with the details of their mental health story.

If there was a really sensitive topic that I said ‘I don't want to be part of that research’, then I would actually ask that [my carer] who might want to be part of it checked with me, ‘Do you mind if I do?’ [Forum Consumer 3]

One consumer felt the carer's experience could not be separated from the consumer story, arguing that ‘the carer story isn't a thing without the consumer… the consumer has more ownership of the story than the carer’. [Forum Consumer 2]. Carers largely felt that they had the right to tell their own side of events as a separate story, over which they have ownership – ‘I feel that we as carers can participate [in research] without having to sort of go to our consumer and say, ‘Now is it alright with you for me to tell my story?’’ [Forum Carer 1].

When consumers and carers were brought together in discussion, some participants maintained strong views regarding a consumer's greater degree of ownership over a shared story – ‘a carer's story will be intrinsically and interdependently attached to the consumer story rather than just being their carer story’ [Forum Consumer 1], whereas others maintained the perspective that carer stories were separate – ‘They're two separate people with two separate stories and the two stories might be polar opposite but there's still a story to tell. And it's the individual who should make the choice’ [Forum Carer 3].

Within this discussion, there was also acknowledgement that the sense of ownership for consumer and carer experiences can be contentious, and that boundaries around story sharing should ideally be discussed to avoid conflict.

…the dynamic we're kind of talking about here, where you have to know within your own relationships ‐‐ that confidence that ‘You have a story, I have a story’ and maybe they're separate. But you have to know that the other person is comfortable with that, or you might cause friction [Forum Consumer 3]

In contrast to the polarized forum discussions, carers and consumers in the interviews held similar views of story ownership. The lived experience story was seen as having both separate and intertwined elements. The caring role was acknowledged as a separate lived experience, and all participants agreed that carers have their own experiences and a story to tell about those experiences. The ownership of this lived experience story depended on the nature of the story a person was trying to tell—‘So the story of the carer's experience as a carer for a person with mental illness, that is absolutely their story’ [Interview Consumer 2].

Participants described the content of the carer story as including experiences with services, the impacts of caring on health and well‐being, and the impact of caring on families. The carer's story did not include speaking on behalf of a consumer's thoughts, experiences or feelings, although it was acknowledged that these boundaries were blurred when a carer was advocating for a consumer with limited capacity.

All participants who talked about the consumer story agreed that consumers have ownership over their story and experiences. The limitations applied to the carer story were also applied to the consumer story; that is, it would be inappropriate for a consumer to speak on behalf of a carer's experiences.

Participants acknowledged that there were also intertwined or shared elements of the lived experience story. When the story being told was shared, it was seen as important to hear both consumer and carer perspectives. Many participants expressed an awareness that there are multiple sides to a story and that people will experience and recall things differently. Though these differences were valuable in a research context, discovering that a person's perspective differs from one's own could have an emotional impact.

…I think it's good to get the carer and the family perspective as well as the consumer perspective, to get the true story, to get what's really happening and how it's impacting on everybody. …so you need every perspective to get the whole picture [Interview Carer 1]

…you just want to think that they are on your side, they're there to help you, they agree with everything… But when you start really realising that no they're not, they're an actual person who has thoughts and feelings of their own, that's when it becomes a bit [pause] it becomes confronting [Interview Consumer 2]

Most participants agreed that a person has a right to tell their own story and to talk about their own experiences but this right was more consistently supported for consumers than for carers. Two consumers felt it would be inappropriate to share a story or to participate in research if the other party in your relationship had specifically asked you not to, particularly when the request came from a consumer. However, both consumer and carer participants noted that being able to prevent another person from talking about their experiences is problematic.

I think it's quite empowering to share your story…and your experiences, and it's useful to research. So I would not like to see one of the pair being able to say to the other one that you can't [Interview Consumer 3]

### 3.2 Communication and education

The topic of *communication and education* received significant discussion in the consumer and combined discussions at the forum. Participants were concerned with making informed choices and commented that in order to give consent, and they had to truly understand the nature of the research and where the data might be published. They felt that it was the responsibility of researchers to inform potential participants about the sensitivities that can arise around privacy issues which may present risks to the consumer‐carer relationship and suggested researchers should educate potential participants about boundaries for participation where this may be a risk.

I think it [would be] nice that it's in the information sheet to be aware or to be knowledgeable to the fact that… your participation in research can impact on others, your family or friends and you may want to consider those relationships [Forum Carer 3]

When asked if specific permission was needed to participate, carers and some consumers suggested that carers should be able to participate independently of the consumer, but acknowledged the value of communication between consumers and carers about research participation.

I think [carers] can validly participate in research if there's some disagreement… it just depends on again the relationship that the person has and that them being aware that if they don't tell the person that they're participating in research, what could eventuate [Forum Consumer 2]

Derived from the forum focus on researchers' responsibility to adequately prepare participants, the importance of informed consent received substantial discussion in the interviews. The need for plain‐language, clear and honest communication in the informed consent process was highlighted.

I think as long as people are transparent about what the research is about, what the aims are and who's conducting it, then it's OK And if they're not transparent then I don't think those people are able to give proper consent, and so then it's not ethical in the first place [Interview Consumer 4]

Similar to the forum, interviewees suggested it would be valuable for researchers to inform participants about potential risks to personal relationships that could result from telling shared stories. They also recommended educating participants about how to discuss research participation within their relationships and how to tell stories safely. A small number of consumers suggested that it may be helpful to have a formal consent process, in which both the consumer and carer agreed to research participation, even if only one member of the pair was participating.

I suppose at the very least it's important to know that that person has had a conversation with the person they're caring for, if it's the carer, regarding what they're OK with being shared and not, in terms of sensitivities and privacy issues. That would probably be my main concern [Interview Consumer 1]

While participants generally agreed that boundaries around telling shared stories were important, the degree to which participants had explicitly discussed these boundaries in their own relationships varied. Participants with experience in advocacy and education roles reported having explicit conversations with the other people involved in the stories they shared as part of their role. However, most participants relied on an implicit understanding between themselves and their family members and friends about what was okay to share.

There's also shared aspects of it [the lived experience story], and I think it's important to negotiate that where the other person's mentioned, and negotiate whether the other person should be de‐identified or whether they want their name associated with the story [Interview Carer 2]

In a research context, most consumers preferred to be informed if their carer was going to participate in a project, particularly if it was focused on consumer experiences. Carers preferred to be informed about consumer participation as a protective measure when they perceived that the person they cared for was vulnerable to potential coercion or psychological harm. Where the focus of research would require a participant to explicitly talk about another person, participants felt the person should be informed and explicitly asked for their consent—‘I'd like to be sort of briefed at least, so that they like at least had my consent to be part of the research’ [Interview Consumer 4]. However, communicating about research within the consumer‐carer relationship was not viewed as essential or beneficial in all circumstances. Some participants felt there was no obligation to communicate about research within their own relationships. Participants tended to agree that consumers do not need to inform their carer when participating in consumer‐focused research. Additionally, several participants acknowledged that the consumer‐carer dynamic varied, and that this may affect the necessity and ease of communication. For example, some carers highlighted the importance of tailoring communication to a consumer's current capacity.

‘And I think that each of those people [consumer and carer] does have a right to decide if they want to go ahead without the other person's knowledge’ [Interview Consumer 2].

## 4 DISCUSSION

The current study findings demonstrate that the lived experience story has both separate and shared elements, and thus, there are potential risks to relationships and to the privacy of non‐participants when conducting mental health research. These risks fall under the categories of social harm and harm to non‐participants, as defined by the Australian National Statement.^18^ There are particular concerns when research involves both the consumer and the carer, but these also warrant consideration when dealing with each group individually. It is the responsibility of researchers and research governance bodies to acknowledge these risks in both information sheets and discussions with potential participants, and to develop resources to facilitate conversations about information‐sharing boundaries.

While both consumers and carers were considered to have ownership over their lived experience story, there were limitations on which elements are personal and which elements are shared. Disclosing the shared elements could compromise the privacy of other people involved in a story. Within consumer‐carer relationships, storytelling boundaries may be implicitly understood or explicitly negotiated. Under certain circumstances, it may be beneficial for researchers to recommend or require explicit discussion of boundaries and research participation within the consumer‐carer dyad.

Consistent with the method implemented by Allam and colleagues 11 in which carers were recruited independently without asking the permission of their consumer ‘pair’, evidence from the current study suggests that where research is focused on the experience of the individual, it is beneficial but not necessary to disclose participation within the consumer‐carer relationship. In these circumstances, the findings suggest that it is not necessary to seek informed consent from a participant's ‘pair’. Instead, researchers could provide information about the risks of telling shared stories and the benefits of discussing information‐sharing boundaries within a relationship, ensuring that participants have sufficient information to understand the potential implications of participating in a research project.^18^ Providing information may facilitate communication without harming participant autonomy. Researchers should also take primary responsibility for protecting the privacy of participants and the people included in their stories.^18^ These findings are consistent with the values and themes of the National Statement.^18^ They also provide both consumer and carer support for the UK MHRN recommendations, which suggest that when research is primarily concerned with the carer experience, it is not necessary to identify carers through consumers or to request permission from consumers to approach their carer; however, consumer permission is considered necessary when the research question is explicitly *focused* on the consumer.^19^

Where research is focused on shared information or the consumer‐carer relationship, a formal informed consent process for both members of the relationship may be desirable. In general, consumer and carer participants felt it would be inappropriate to talk directly about another person's experiences without their explicit consent, particularly when that person was a consumer. The National Statement indicates that where research involves properly interested parties, which in this case may include family members and friends, all interested parties should be involved in planning the research.^18^ In these cases, a consent process as recommended by the MHRN 19 could be implemented to ensure that both parties in the relationship were aware of the research study and that information‐sharing boundaries had been discussed.

The forum data suggested that the potential risks of harm to non‐participants resulting from sharing a lived experience story justify implementing these communication and consent processes. Forum consumers felt particular vulnerability within the consumer‐carer dyad due to the risk that their sensitive personal information could be shared by a carer. While this perceived vulnerability was not raised by consumers in the interviews, it was acknowledged that discovering a person's perspective differs from one's own could have an emotional impact. This suggests that sharing personal information can pose a risk to privacy or reputation for non‐participant consumers or cause discomfort. The likelihood and severity of the risks of storytelling will vary between studies, and further research evidence may be required to appropriately gauge these factors.^18^ For example, the findings of this study do not provide specific guidance for working with carers of consumers who disagree with their diagnosis, despite the use of discussion prompts related to this issue. Future in‐depth research may clarify the ethical dilemmas that could arise when working with this population. Within the limits of national guidelines, a balanced approach to managing risks is recommended, implementing as few safeguards as possible, yet as many as necessary.^20^

The conclusions that can be drawn from the findings of the present study are limited by the small number of mostly female participants recruited from a restricted geographical area. While human research practices are based on the same founding ethical principles internationally, and procedural approaches to ethical review are similar in Australia, Canada and the United States,^16^, ^17^, ^18^, ^26^ some of the research experiences of participants may be specific to the Australian context. In future, the authors intend to use these findings to begin a broader consultation and co‐creation process with carers, consumers, researchers and other relevant stakeholders in Australia to develop guidelines to supplement the National Statement 18 and support the ethical and safe conduct of mental health research involving carers.

## 5 CONCLUSION

Conducting research involving mental health carers and consumer‐carer relationships raises ethical issues related to story ownership, including risks of harm to participants' personal relationships and to the privacy or reputation of non‐participants. Research practice in these areas may require a different approach to research involving mental health consumers or the general population. In particular, it may be necessary to facilitate negotiation of information‐sharing boundaries within relationships and the safe and confidential telling of shared stories. When implementing ethical safeguards, it is important to maintain participant autonomy and ensure measures are respectful and acceptable to the community concerned.^20^

Carers contribute a unique set of lived experiences, perspectives and agendas to research and their role in mental health research is increasing.^4^, ^5^, ^6^, ^7^, ^8^, ^9^, ^10^, ^11^, ^12^, ^13^, ^14^, ^15^ It is important to continue to facilitate the participation of mental health carers so their experiences can inform our evidence base and subsequently inform the development of evidence‐based clinical practice and policy. The results of the present research can contribute to the development of guidelines for the ethical and safe conduct of research involving carers, supplementing existing national ethics guidelines and facilitating quality mental health research in this specialized area.

## CONFLICT OF INTEREST

The authors declare no conflict of interest.

## Data Availability

Due to ethical restrictions, the research data are not shared.
